# Tumor Clusterin Expression at Diagnosis Is Associated with Residual Disease Burden After Neoadjuvant Chemotherapy in Breast Cancer

**DOI:** 10.3390/jcm15197557

**Published:** 2026-09-29

**Authors:** Marilina García-Aranda, Desirée Martín-García, Irene Zarcos-Pedrinaci, Diego Pérez, Ioana-Cristina Tugulea-Teodorovici, Sergio A. Rodríguez-Lobalzo, Francisco Rivas-Ruiz, Francisco-Javier Martínez-Barrios, Gema Civantos, Marta Legeren, Nicolás Gallego-Pena, Esteban Nogales, María Luisa Hortas, Raquel Barco, Cristina Quero, Maximino Redondo

**Affiliations:** 1 Telecommunication Research Institute (TELMA), E.T.S. Ingeniería de Telecomunicación and Mobilenet, University of Málaga, 29071 Malaga, Spain; 2 Red de Investigación en Servicios de Salud en Enfermedades Crónicas (REDISSEC) and Red de Investigación en Cronicidad, Atención Primaria y Promoción de la Salud (RICAPPS), 29603 Marbella, Spain; 3 Instituto de Investigación Biomédica de Málaga y Plataforma en Nanomedicina—IBIMA Plataforma BIONAND, 29590 Malaga, Spain; 4 Surgical Specialties, Biochemistry and Immunology Department, Faculty of Medicine, University of Málaga, 29010 Malaga, Spain; 5 Department of Oncology, Hospital Universitario Costa del Sol, Autovía A-7, Km 187, 29603 Marbella, Spain; 6 Pathological Anatomy Service, Hospital Universitario Costa del Sol, Autovía A-7, Km 187, 29603 Marbella, Spain; 7 Research and Innovation Unit, Hospital Universitario Costa del Sol, Autovía A-7, Km 187, 29603 Marbella, Spain; 8 Department of Pathology, Virgen del Rocío University Hospital, 41013 Sevilla, Spain; 9 Department of Oncology, San Cecilio University Hospital, 18016 Granada, Spain; 10 Instituto de Investigación Biosanitaria de Granada (ibs.Granada), Av. de Madrid, 15, Beiro, 18012 Granada, Spain; 11 Department of Oncology, Virgen Macarena University Hospital, 41009 Sevilla, Spain; 12 Clinical Laboratories Area, Hospital Universitario Costa del Sol, Autovía A-7, Km 187, 29603 Marbella, Spain

**Keywords:** clusterin, breast cancer, neoadjuvant chemotherapy, residual cancer burden, pathological response, immunohistochemistry

## Abstract

**Objectives**: Clusterin (CLU) has been implicated in cell survival and treatment resistance, but its relationship with pathological response to neoadjuvant chemotherapy in breast cancer remains unclear. We evaluated CLU expression in routine diagnostic specimens as a biomarker of pathological response and long-term outcomes. **Methods**: This multicenter study included 152 patients with breast cancer treated with neoadjuvant chemotherapy. CLU expression was assessed by immunohistochemistry and analyzed across four predefined expression categories. Associations with pathological complete response (pCR), residual cancer burden (RCB), and long-term outcomes were evaluated using logistic and ordinal regression and time-to-event analyses. Multivariable ordinal regression assessed the independent association between CLU expression and RCB. **Results**: CLU was evaluable in 131 tumors, with high interobserver reproducibility (97.5% agreement; weighted κ = 0.932). Increasing CLU expression was inversely associated with histological grade (ρ = −0.29; *p* = 0.003). Among 73 patients with RCB assessment, increasing CLU expression was associated with greater residual cancer burden (OR per category increase = 1.53, 95% CI 1.01–2.31; *p* = 0.044), whereas low (<50%) CLU expression was associated with higher odds of achieving RCB 0 (Firth OR = 5.23, 95% CI 1.53–17.96; *p* = 0.008). Among 105 patients evaluable for pCR, pCR was more frequent in low- than high-CLU tumors (28.6% vs. 12.5%; OR = 2.80, 95% CI 1.02–7.65; Fisher’s exact *p* = 0.051). CLU expression was not associated with disease-free survival, distant recurrence-free survival or overall survival. **Conclusions**: CLU expression at diagnosis shows an ordered association with residual disease burden after neoadjuvant chemotherapy. This association was not independent of clinical stage and subtype, and CLU therefore warrants further validation as a response-associated tissue biomarker rather than as an independent predictor.

## 1. Introduction

Breast cancer is the most frequently diagnosed malignancy among women worldwide and remains a leading cause of cancer-related mortality despite major advances in systemic therapy [1]. Neoadjuvant chemotherapy has become a standard treatment strategy for patients with locally advanced and selected early-stage breast cancer, providing not only tumor downstaging but also a unique opportunity to evaluate tumor sensitivity to systemic treatment in vivo [2]. Optimizing treatment is particularly relevant given that, beyond oncological outcomes, breast cancer and its treatment can have persistent functional, psychosocial and socioeconomic consequences, including limitations in daily activities, employment difficulties, financial burden, and unmet supportive care needs [3].

In this context, tools that help tailor neoadjuvant treatment from diagnosis, avoiding ineffective treatment and its associated burden, are of particular clinical value. Pathological response after neoadjuvant chemotherapy has important prognostic implications and is increasingly used as an intermediate endpoint in clinical trials and treatment decision-making [4]. However, patients with apparently similar clinicopathological characteristics frequently exhibit markedly different responses to the same chemotherapy regimens. Despite considerable research efforts, currently available clinicopathological and molecular biomarkers remain insufficient to accurately predict individual response before treatment initiation, highlighting the need for novel response-associated biomarkers suitable for routine clinical practice [5].

Pathological complete response (pCR) is the most widely used endpoint evaluating response to neoadjuvant chemotherapy, particularly in aggressive breast cancer subtypes. Nevertheless, its binary definition does not fully capture the biological spectrum of residual disease. Residual cancer burden (RCB), by incorporating primary tumor dimensions, tumor cellularity and nodal involvement, provides a more comprehensive quantification of treatment response and has demonstrated robust prognostic value across breast cancer subtypes [6,7]. Biomarkers associated with RCB may therefore offer a more informative assessment of tumor chemosensitivity than those evaluated exclusively against pCR. Current research is increasingly moving towards multimodal predictive strategies integrating clinicopathological, molecular and imaging biomarkers to improve response prediction [5].

Clusterin (CLU) is a highly conserved glycoprotein involved in cellular stress responses, protein homeostasis, and apoptosis regulation [8,9,10]. Increasing experimental evidence indicates that CLU expression is induced by chemotherapy-associated cellular stress, promoting tumor cell survival through inhibition of apoptosis and activation of pro-survival signaling pathways [8,10,11,12]. Consequently, CLU has emerged as a potential mediator of treatment resistance in several solid tumors, including breast cancer [8,10].

Despite the biological rationale, the clinical value of pretreatment CLU expression as a response-associated biomarker to neoadjuvant chemotherapy remains insufficiently characterized. Previous studies have reported an association between increased CLU expression and poorer response to neoadjuvant treatment in breast cancer [13,14]; however, these investigations are limited by relatively small, single-center cohorts, restricted patient populations, or evaluation of pathological response using binary endpoints alone. Whether CLU expression assessed in routine diagnostic biopsy specimens is associated with the extent of residual disease after treatment, as measured by RCB, has not been adequately explored.

Therefore, the aim of this multicenter study was to investigate whether CLU expression assessed by immunohistochemistry in routine pretreatment diagnostic breast biopsy specimens from patients with breast cancer treated with neoadjuvant chemotherapy is associated with pathological response to neoadjuvant chemotherapy, with particular emphasis on RCB as a biologically informative measure of treatment response. In addition, we evaluated the relationship between CLU expression and long-term oncological outcomes to determine whether its potential clinical utility lies in being associated with treatment response, prognosis, or both. By evaluating CLU across the full spectrum of RCB rather than relying exclusively on binary pCR, this study aimed to determine whether pretreatment CLU expression captures clinically relevant heterogeneity in pathological response that may otherwise remain undetected.

## 2. Materials and Methods

### 2.1. Study Design, Scope, and Period of Study

This retrospective biomarker study was conducted within a prospective, multicenter cohort of 152 patients with breast cancer treated with neoadjuvant chemotherapy at four hospitals within the Andalusian Public Health System, Spain: Costa del Sol University Hospital (Marbella), Virgen del Rocío University Hospital and Virgen Macarena University Hospital (Seville), and San Cecilio University Hospital (Granada). Patients were diagnosed between 2006 and 2019 and received anthracycline- and/or taxane-based neoadjuvant chemotherapy according to routine clinical practice.

The present study investigated the association between pretreatment clusterin (CLU) expression in diagnostic breast tumor samples and pathological response to neoadjuvant chemotherapy, with long-term oncological outcomes evaluated as secondary endpoints. Clinical and pathological data were obtained from the prospective multicenter cohort, whereas CLU expression was retrospectively assessed by immunohistochemistry in available diagnostic biopsies.

### 2.2. Inclusion and Exclusion Criteria

Eligible participants were adults diagnosed with stage I–IV breast cancer who received neoadjuvant cytotoxic chemotherapy between 2006 and 2019 at one of the participating centers and provided study-specific informed consent. Patients with clinical stage IV disease at diagnosis were included when they had received primary systemic (neoadjuvant) chemotherapy followed by surgery within routine multidisciplinary practice, and were thus managed within the same neoadjuvant pathway as the rest of the cohort. Patients were required to have available clinical and pathological information and a pretreatment diagnostic breast biopsy suitable for biomarker assessment. Patients who had received systemic anticancer therapy or radiotherapy before collection of the diagnostic biopsy were excluded.

### 2.3. Data Collection and Variables

Clinical and pathological data were collected from medical records and institutional databases at each participating center by trained reviewers using a standardized data collection protocol. Patients were prospectively and consecutively identified at each center, whereas the present biomarker analysis was performed retrospectively using available diagnostic biopsy. Pretreatment diagnostic biopsy specimens were retrieved from residual diagnostic material or from samples deposited in the Biobank of the Andalusian Public Health System, in accordance with the corresponding informed consent and applicable regulations. Tissue processing and storage were performed by the pathology departments of the participating hospitals and the Biobank according to standard procedures.

Collected clinicopathological variables included age at diagnosis, family history of breast cancer or BRCA mutation, menopausal status, diagnostic context (clinical presentation or radiological screening), histological type and grade, vascular invasion, clinical stage, estrogen receptor (ER), progesterone receptor (PR) and HER2 status, Ki-67 proliferation index, tumor-infiltrating lymphocytes (TILs), and breast cancer immunophenotype. Treatment and follow-up variables included type of breast and axillary surgery, systemic and locoregional treatments, pathological response to neoadjuvant chemotherapy, local and distant recurrence, vital status, date of last follow-up, and date of death where applicable.

Pathological response was assessed according to the system routinely used at each participating center and time period, including the Miller–Payne grading system and residual cancer burden (RCB). For the present study, RCB was analyzed according to its four standard categories (RCB 0, I, II, and III). Pathological complete response (pCR) was defined as the absence of residual invasive disease in both the breast and axillary lymph nodes. Accordingly, pCR corresponded to RCB 0 or, for cases assessed using the Miller–Payne system, Miller–Payne grade 5 together with complete axillary response. For secondary analyses, CLU expression was also dichotomized as low (<50%) or high (≥50%).

Long-term oncological outcomes included disease-free survival (DFS), distant recurrence-free survival (DRFS), and overall survival (OS). DFS was defined as the time from histological diagnosis to the first occurrence of local recurrence, distant recurrence, or death from any cause. DRFS was defined as the time from histological diagnosis to distant recurrence or death from any cause, and OS as the time from histological diagnosis to death from any cause. Patients without the corresponding event were censored at the last date at which their event status was ascertainable. Patients with a documented event for which an event date could not be ascertained were excluded from the corresponding time-to-event analysis. Five-year DFS, DRFS, and OS estimates were additionally calculated as prespecified time-point outcomes.

### 2.4. Immunohistochemical Assessment of CLU Expression

Pretreatment diagnostic tumor specimens were formalin-fixed and paraffin-embedded (FFPE) according to standard pathological procedures. Routine assessment of estrogen receptor (ER), progesterone receptor (PR), HER2, and Ki-67 was performed as part of the diagnostic pathological evaluation at each participating center.

Stromal tumor-infiltrating lymphocytes (sTILs) were assessed on 4–5 μm hematoxylin and eosin-stained sections from diagnostic FFPE specimens according to the recommendations of the International TILs Working Group 2014 [15]. The evaluable stromal area was defined within the borders of the invasive tumor, excluding tumor margins, areas surrounding ductal carcinoma in situ, and normal lobules. Mononuclear inflammatory cells within the tumor stroma were evaluated, excluding granulocytes and other polymorphonuclear leukocytes, and sTILs were recorded as the percentage of stromal area occupied by mononuclear inflammatory cells (0–100%).

CLU immunohistochemistry was performed centrally at the Pathology Laboratory of Costa del Sol University Hospital using a monoclonal antibody against the CLU α-chain (clone 41D; Upstate-Millipore, Watford, UK) at a 1:400 dilution on an automated Ventana DISCOVERY ULTRA staining platform (Roche Diagnostics, Basel, Switzerland), following the previously described protocol [16]. Internal positive controls were provided by constitutive CLU staining in lymphocytes, while appropriate negative controls were included in each staining run.

Anonymized CLU-stained sections were independently evaluated by two pathologists blinded to clinicopathological characteristics, treatment response, and survival outcomes. For each case, CLU staining was assessed across all available tumor tissue in the biopsy sections. Slides were first reviewed at low magnification (×4) to identify tumor areas and estimate the overall extent of staining, and the proportion of positive tumor was then evaluated at ×10; the subcellular staining pattern was confirmed at ×20–×40. Positive CLU expression was defined as cytoplasmic or membranous staining in tumor cells and/or stromal immunoreactivity involving ≥10% of the tumor-associated stroma. No case showed isolated stromal positivity in the absence of tumor-cell staining. Stromal staining extent and intensity were not recorded separately from tumor-cell staining. Each case was classified according to the percentage of tumor showing positive CLU staining. CLU expression was scored prospectively by the study pathologists according to a pre-specified protocol, based on the proportion of positive tumor cells, and categorized as negative (<10%), 10–50%, 50–90%, or >90%. The <10% threshold for negativity was consistent with our group’s previous clusterin immunohistochemical assessment [16]. Discordant cases were reviewed by a third pathologist to reach a final classification. Interobserver agreement was assessed using percentage agreement and weighted Cohen’s κ.

### 2.5. Statistical Analysis

Continuous variables were summarized as mean and standard deviation (SD) or median and interquartile range (IQR), as appropriate, and categorical variables as absolute and relative frequencies. Percentages were calculated using the number of evaluable patients for each variable as the denominator. Missing data were not imputed, and analyses were performed using available cases.

Associations between CLU expression and clinicopathological characteristics were assessed according to variable type. Correlations between ordinal CLU expression and continuous or ordinal variables were evaluated using Spearman’s rank correlation coefficient. Associations with categorical variables were examined using the χ^2^ test or Fisher’s exact test, as appropriate. Interobserver reproducibility of CLU assessment was evaluated using percentage agreement and weighted Cohen’s κ with 95% confidence intervals (CIs).

The association between CLU expression and pathological response to neoadjuvant chemotherapy was evaluated using complementary approaches. Pathological complete response (pCR) was analyzed as a binary outcome using logistic regression, with odds ratios (ORs) and 95% confidence intervals (CIs) reported. CLU expression was evaluated both across the four expression categories and, in a secondary analysis, dichotomized as low (<50%) versus high (≥50%). Given the limited number of pCR events, Fisher’s exact test was used to compare pCR frequencies between low and high CLU expression groups. A Cochran–Armitage trend test was used to assess the trend in pCR across the four ordered CLU expression categories. The incremental contribution of CLU to the multivariable model was assessed by comparing nested models with and without CLU using a likelihood-ratio test.

Among patients with available RCB assessment, the ordered association between CLU expression and increasing RCB category (RCB 0, I, II, and III) was evaluated using ordinal logistic regression, with CLU entered as an ordinal variable. The proportional-odds assumption was assessed before interpretation of the model. The overall RCB distribution was additionally compared between low and high CLU expression groups. Given the limited number of RCB 0 events, its association with dichotomized CLU expression was evaluated using Firth penalized logistic regression to reduce small-sample bias.

A parsimonious multivariable ordinal logistic regression model was used to examine whether the association between CLU expression and RCB persisted after adjustment for clinically relevant covariates. The model included CLU expression as an ordinal variable, age as a continuous variable, clinical stage grouped as I–II versus III–IV, and breast cancer subtype categorized as hormone receptor-positive/HER2-negative (HR+/HER2−), HER2-positive, or triple-negative breast cancer (TNBC). The number of covariates was restricted in view of the available sample size and number of outcome observations. Histological grade was excluded from the primary model because of missing data and was included in a sensitivity analysis.

Long-term oncological outcomes were analyzed using time-to-event methods. Kaplan–Meier estimates were used to describe DFS, DRFS, and OS, and survival distributions were compared using the log-rank test where appropriate. Cox proportional-hazards regression was used to estimate hazard ratios (HRs) and 95% CIs, with CLU modeled ordinally across its four expression categories. Five-year survival estimates were derived from the corresponding Kaplan–Meier curves. Crude recurrence and mortality frequencies were considered descriptive and analyzed separately from time-to-event outcomes. Exploratory subgroup analyses were performed according to breast cancer immunophenotype; given the limited sample size within individual subgroups, these analyses were considered hypothesis-generating.

All statistical tests were two-sided, and *p* values < 0.05 were considered statistically significant. Analyses were performed using R version 4.1.1.

**Figure 1:**
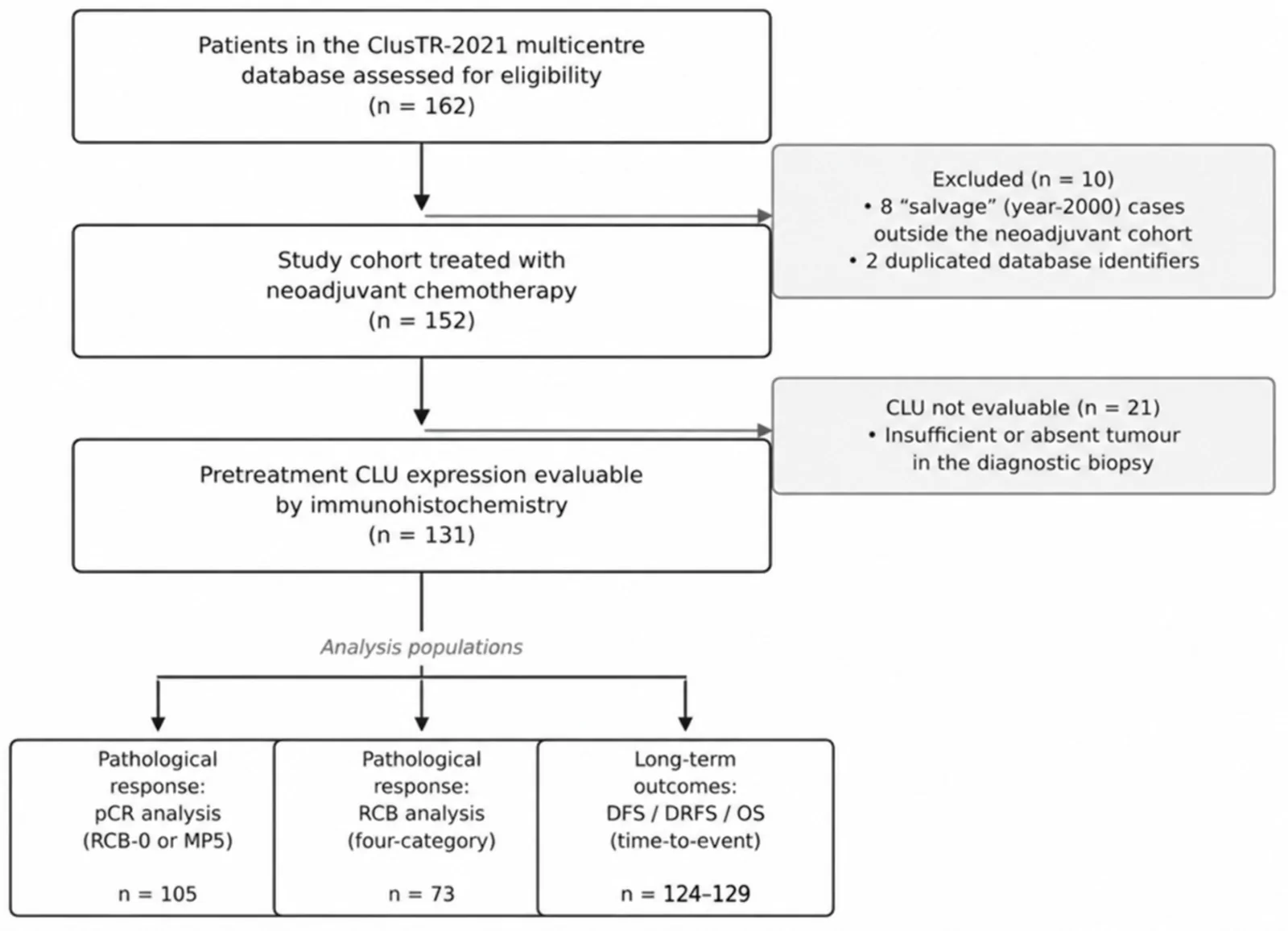
Patient flow through the study. Of 162 patients in the multicenter database, 10 were excluded (8 year-2000 salvage cases outside the neoadjuvant cohort and 2 duplicated database identifiers), yielding a study cohort of 152 patients treated with neoadjuvant chemotherapy. Pretreatment clusterin (CLU) expression was evaluable by immunohistochemistry in 131 patients. Analysis populations differed across endpoints according to data availability: pathological complete response (pCR; *n* = 105), four-category residual cancer burden (RCB; *n* = 73), and long-term time-to-event outcomes (*n* = 124–129 depending on endpoint and ascertainable event dates). CLU, clusterin; DFS, disease-free survival; DRFS, distant recurrence-free survival; MP5, Miller-Payne grade 5; OS, overall survival; pCR, pathological complete response; RCB, residual cancer burden.

The flow of patients from the source database to the individual analysis populations is summarized in Figure 1.

## 3. Results

### 3.1. Characteristics of the Study Cohort

A total of 152 patients with breast cancer treated with neoadjuvant chemotherapy were included in the study. The mean age at diagnosis was 50.2 years (SD 11.1), and 55.3% of patients were pre- or perimenopausal. Most tumors were diagnosed following clinical presentation (84.2%) and were of invasive ductal histology (90.8%). Among tumors with available histological grading, grade III was the most frequent (51.7%). Luminal B was the most common immunophenotype (46.1%), followed by basal-like disease (22.4%). Baseline clinicopathological and molecular characteristics of the cohort are summarized in Table 1.

Baseline clinicopathological characteristics according to inclusion in the CLU-RCB analysis are shown in Supplementary Table S1. No significant differences were observed in clinical stage, histological grade, ER or PR status, or Ki-67. However, patients included in the RCB analysis differed in breast cancer subtype distribution and were more frequently diagnosed in later years. A marked center-dependent distribution was also observed, consistent with the progressive and non-synchronous implementation of RCB across participating institutions during the study period.

### 3.2. CLU Expression Across Clinicopathological Characteristics

**Figure 2:**
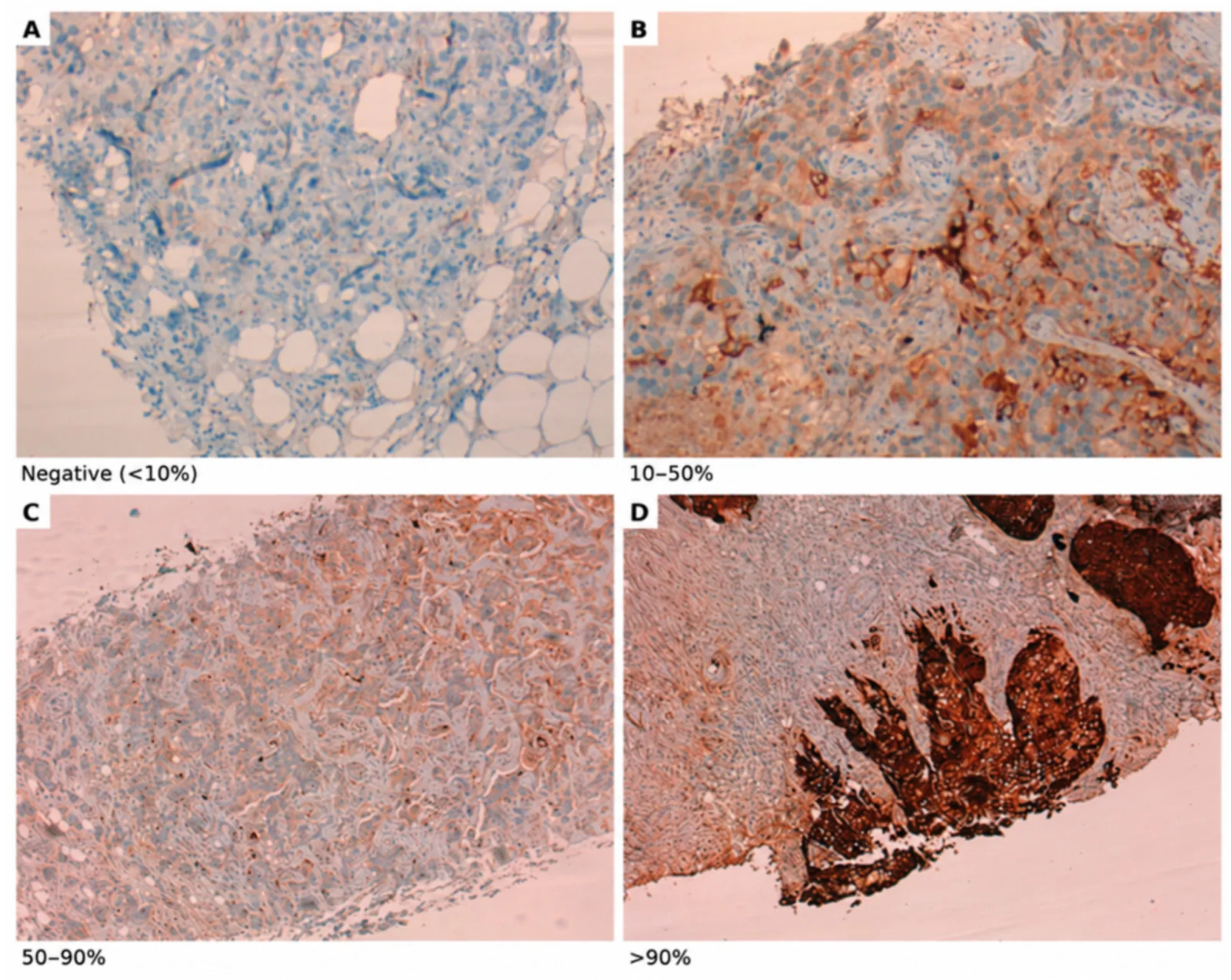
Representative immunohistochemical staining of clusterin (CLU) in diagnostic breast cancer biopsies across expression categories. (**A**) CLU-negative (<10% positive cells); (**B**) 10–50%; (**C**) 50–90%; (**D**) >90% positive cells. Brown staining indicates CLU immunoreactivity, with blue hematoxylin counterstaining. Positive CLU staining was defined as cytoplasmic or membranous immunoreactivity in tumor cells; stromal immunoreactivity involving ≥10% of the tumor-associated stroma was also recorded, although no case showed isolated stromal positivity in the absence of tumor-cell staining. Anti-CLU monoclonal antibody against the CLU α-chain (clone 41D; Upstate-Millipore, Watford, UK, 1:400 dilution). Magnification ×40 (**A**–**C**), ×20 (**D**).

CLU expression was evaluable in 131 patients and showed substantial intertumoral heterogeneity. 31 (23.7%) were CLU-negative, 35 (26.7%) showed expression in 10–50% of tumor cells, 26 (19.8%) in 50–90%, and 39 (29.8%) in >90%. CLU assessment showed high interobserver reproducibility, with 97.5% agreement between pathologists and weighted κ of 0.932 (95% CI, 0.890–0.974) (Figure 2).

Across baseline clinicopathological and molecular characteristics, CLU expression was independent of age, menopausal status, family history, diagnostic context, vascular invasion, estrogen- and progesterone-receptor status, HER2 status, clinical stage, immunophenotype, sTILs and Ki-67 category (all *p* > 0.05). Histological grade was the only variable significantly associated with CLU expression, showing an inverse ordinal relationship (Spearman ρ = −0.29, *p* = 0.003). Grade III tumors showed a higher proportion of CLU-negativity (31.4%) and a lower proportion of >90% CLU expression. Ki-67, evaluated as a continuous variable, showed a weak inverse correlation in the same direction (ρ= −0.19, *p* = 0.073).

### 3.3. CLU Expression and Pathological Response to Neoadjuvant Chemotherapy

**Figure 3:**
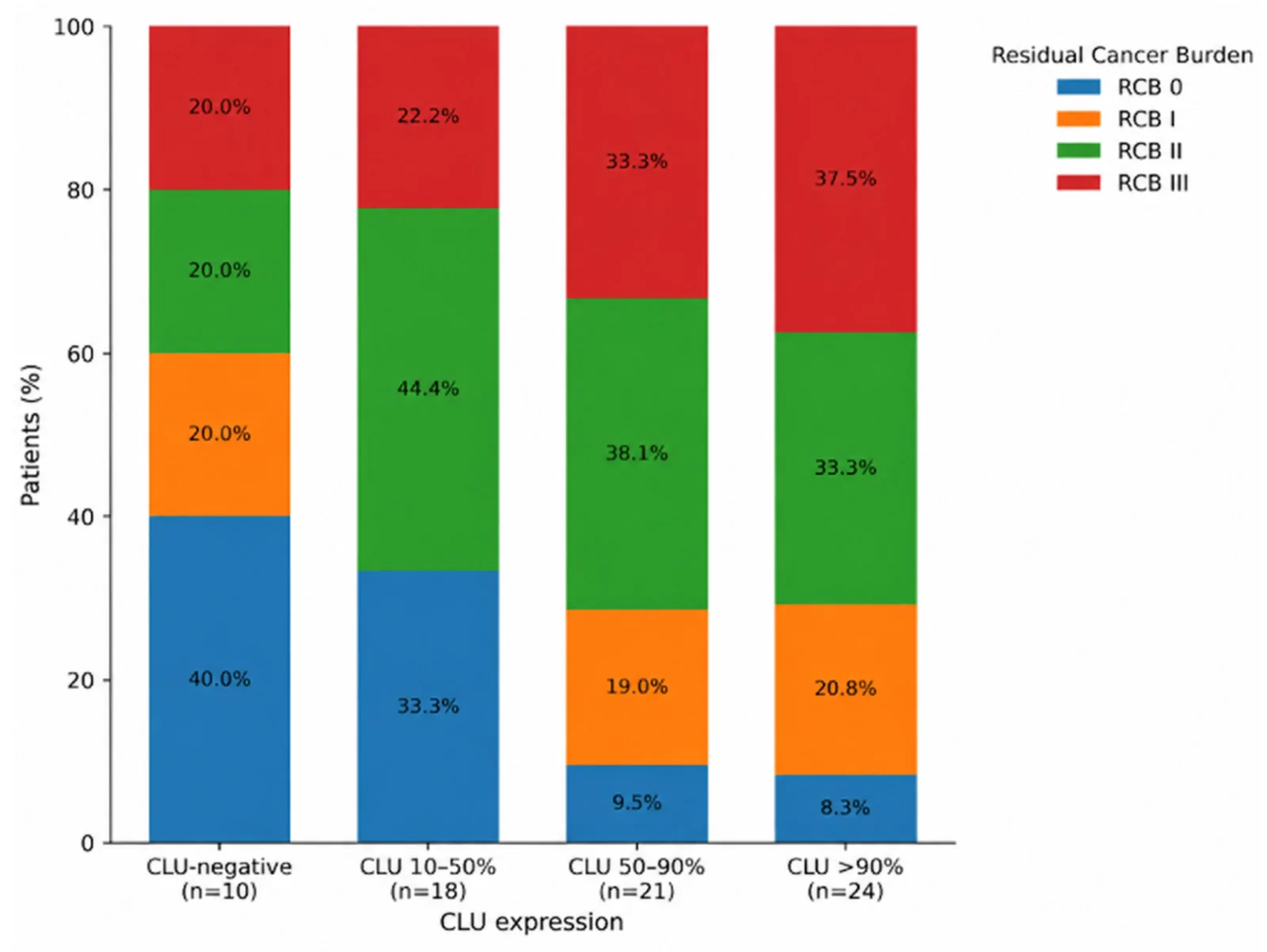
Distribution of residual cancer burden according to CLU expression following neoadjuvant chemotherapy. Stacked bars show the proportion of patients classified as RCB 0, I, II, or III across the four CLU expression categories among patients with available RCB assessment (*n* = 73). Increasing CLU expression was associated with a progressive shift toward greater residual disease burden, with RCB 0 decreasing from 40.0% in CLU-negative tumors to 8.3% in tumors with >90% CLU expression, whereas RCB III increased from 20.0% to 37.5%, respectively. Ordinal logistic regression showed higher odds of belonging to a greater RCB category with increasing CLU expression (OR per category increase = 1.53, 95% CI 1.01–2.31; *p* = 0.044).

Pathological response to neoadjuvant chemotherapy was evaluated according to CLU expression. Among the 73 patients with quantitative RCB assessment, increasing CLU expression showed an ordered association with greater residual disease burden. The proportion achieving RCB 0 decreased from 40.0% in CLU-negative tumors and 33.3% in tumors with 10–50% CLU expression to 9.5% and 8.3% in tumors with 50–90% and >90% expression, respectively, whereas RCB III increased from 20.0% and 22.2% to 33.3% and 37.5% across the same categories. Ordinal logistic regression showed that each increase in CLU expression category was associated with higher odds of belonging to a greater RCB category (OR = 1.53, 95% CI 1.01–2.31; *p* = 0.044). In a sensitivity analysis excluding patients with stage IV disease, the ordered association between CLU expression and RCB was maintained (OR per category increase 1.70, 95% CI 1.12–2.60; *p* = 0.014). The overall RCB distribution differed significantly between low (<50%) and high (≥50%) CLU expression (χ^2^ = 9.51; *p* = 0.023), and low CLU expression was associated with markedly higher odds of achieving RCB 0 in Firth penalized logistic regression (OR = 5.23, 95% CI 1.53–17.96; *p* = 0.008) (Table 2; Figure 3).

Consistent with these findings, pathological complete response (pCR) was more frequent in low- than high-CLU tumors among the 105 patients evaluable for pCR (28.6% vs. 12.5%; OR = 2.80, 95% CI 1.02–7.65). This difference approached statistical significance on exact testing (Fisher *p* = 0.051), whereas no significant linear trend was observed across the four CLU expression categories (Cochran–Armitage *p* = 0.075).

To assess whether the association between CLU expression and residual disease burden was independent of established clinical factors, a multivariable ordinal logistic regression model was fitted in the 68 patients with complete data for CLU expression, RCB, age, clinical stage, and breast cancer subtype; covariates were prespecified based on established clinical relevance and potential confounding rather than univariable statistical significance. The association between increasing CLU expression and higher RCB category was attenuated and no longer statistically significant (adjusted OR = 1.34, 95% CI 0.86–2.07; *p* = 0.194). In a sensitivity analysis additionally adjusting for histological grade (*n* = 58), the CLU association remained directionally consistent but non-significant (aOR = 1.31, *p* = 0.280). Advanced clinical stage (III–IV vs. I–II) remained independently associated with greater residual disease burden (aOR = 4.10, 95% CI 1.50–11.23; *p* = 0.006), as did the HR+/HER2− subtype compared with HER2-positive disease (aOR = 10.42, 95% CI 3.22–33.79; *p* < 0.001), whereas no significant difference was observed for triple-negative tumors (aOR = 1.72, 95% CI 0.42–7.04; *p* = 0.448). Addition of CLU to the multivariable model, including age, clinical stage, and breast cancer subtype, did not significantly improve model fit (likelihood ratio χ^2^(1) = 1.70, *p* = 0.192; AIC 164.57 without CLU vs. 164.87 with CLU). Thus, although the direction of the CLU–RCB association was maintained after adjustment, its magnitude was attenuated and statistical significance was lost (Table 2).

Overall, lower CLU expression was associated with a more favorable pathological response profile, particularly with lower residual disease burden in the RCB-assessed subset. Although the direction of the CLU–RCB association was maintained after adjustment for age, clinical stage, and breast cancer subtype, its magnitude was attenuated and statistical significance was lost. Exploratory analyses stratified by immunophenotype did not identify statistically significant associations between CLU expression and pathological response within individual subgroups, although these analyses were limited by small sample sizes.

### 3.4. CLU Expression and Long-Term Oncological Outcomes

Long-term oncological outcomes were analyzed according to CLU expression using time-to-event analyses. After a median follow-up of 76.7 months (IQR 63.7–98.1; reverse Kaplan–Meier method), 50 distant recurrences and 51 deaths were recorded in the cohort.

In Cox proportional-hazards models with CLU modeled ordinally across its four expression categories, CLU expression was not associated with disease-free survival (DFS; HR per category increase = 0.95, 95% CI 0.73–1.23; *p* = 0.702), distant recurrence-free survival (DRFS; HR = 0.88, 95% CI 0.67–1.16; *p* = 0.359), or overall survival (OS; HR = 1.04, 95% CI 0.76–1.42; *p* = 0.794) (Table 3).

## 4. Discussion

In this multicenter cohort, higher CLU expression in routine diagnostic biopsy specimens was associated with greater residual disease burden following neoadjuvant chemotherapy. These findings support further evaluation of CLU as a candidate tissue response-associated biomarker for pretreatment stratification. Although CLU was not associated with long-term oncological outcomes, its potential value lies in being associated with treatment response rather than prognosis.

Few studies have specifically evaluated CLU as a predictor of neoadjuvant chemotherapy response in breast cancer, and these have been limited by small, single-center cohorts. In 72 patients with locally advanced disease, Niu et al. found that high pretreatment tumor secretory CLU was associated with resistance to anthracycline-based (CAF) chemotherapy [13], whereas Wang et al., in 72 triple-negative tumors, localized the predictive signal to the tumor-associated stroma, where low stromal CLU predicted a better pathological response [14]. Unlike Wang et al., who specifically evaluated stromal CLU expression in triple-negative breast cancer, our scoring integrated both tumor-cell and stromal staining. However, no case showed isolated stromal positivity in the absence of tumor-cell staining, indicating that stromal staining did not independently determine CLU classification in our cohort. Consequently, our study was not designed to assess the relative predictive contribution of epithelial and stromal CLU expression. Nevertheless, the concordant direction across independent cohorts (greater CLU, poorer response) reinforces the link between CLU and reduced sensitivity to neoadjuvant chemotherapy, and indicates that the cellular source of CLU warrants dedicated evaluation.

Beyond these individual studies, recent reviews have consolidated CLU as a multifunctional stress-response protein involved in tumor progression, immune modulation and therapeutic resistance across several solid malignancies. Although most evidence remains preclinical, these observations provide additional biological support for the present findings and reinforce the rationale for evaluating CLU as a response-associated biomarker in breast cancer [8,17].

The observed association is biologically plausible given the established role of secretory CLU as a stress-induced cytoprotective chaperone involved in apoptosis regulation, protein homeostasis and treatment resistance. Experimental studies have shown that CLU is induced by chemotherapy-associated cellular stress and functions as a cytoprotective molecular chaperone that inhibits apoptosis, maintains protein homeostasis and activates pro-survival pathways, including AKT and NF-κB. More recently, CLU has also been implicated in epithelial–mesenchymal transition, metastatic dissemination and therapy resistance through complex interactions with multiple signaling networks [8,9,11,12,18]. Consistent with these mechanisms, increased CLU expression has been associated with reduced sensitivity to chemotherapy in breast cancer [10,13,19]. Our findings extend this evidence by showing that higher CLU expression in diagnostic biopsy specimens is associated with greater residual disease burden after treatment, supporting the concept that CLU expression may reflect a cytoprotective tumor state associated with reduced sensitivity to neoadjuvant chemotherapy. These observations were also consistent with our previous findings showing increased CLU expression in recurrent and metastatic breast carcinomas compared with the corresponding primary tumors [16], suggesting that CLU may participate in tumor progression while also contributing to treatment adaptation.

CLU exists as two functionally distinct isoforms: a secretory isoform (sCLU), predominantly localized in the cytoplasm and associated with cytoprotective and anti-apoptotic functions, and a nuclear isoform (nCLU), associated with pro-apoptotic signaling [9]. In our samples, CLU immunoreactivity showed a predominantly cytoplasmic and membranous pattern, consistent with the expected localization of sCLU, whereas nuclear staining was considered negative. This staining pattern therefore supports the interpretation that our findings predominantly reflect the cytoprotective, secretory form of CLU. Nevertheless, because the 41D antibody recognizes the CLU α-chain and has not been established as isoform-specific, definitive attribution to sCLU would require isoform-specific approaches.

A further consideration is the clinical profile of the study population. All patients received neoadjuvant chemotherapy and therefore represent a cohort for biologically aggressive breast cancers requiring systemic treatment before surgery. Even within this high-risk population, substantial intertumoral heterogeneity in CLU expression remained, allowing pathological response to be discriminated across increasing levels of CLU expression and indicating that CLU captures biological heterogeneity beyond conventional clinicopathological markers.

An important methodological strength is that pathological response was analyzed according to the original scoring systems used in routine clinical practice rather than by collapsing Miller–Payne and RCB into a single harmonized outcome. The most informative association emerged when pathological response was evaluated using RCB rather than pCR. Unlike the binary definition of pathological complete response, RCB captures the full spectrum of residual disease and has consistently demonstrated stronger prognostic discrimination and greater biological resolution than binary pathological endpoints across breast cancer subtypes [6], providing a more biologically informative measure of treatment response and reinforcing the suitability of RCB as the preferred endpoint for evaluating biomarkers of chemosensitivity. Preserving this granularity allowed the identification of an ordered relationship between CLU expression at diagnosis and residual tumor burden that would probably have been diluted had pathological response been reduced to a dichotomous endpoint alone. These findings also highlight the value of analyzing biologically informative response endpoints rather than relying exclusively on pCR when evaluating candidate response-associated biomarkers.

Although the association between CLU expression and RCB was attenuated after adjustment for clinical stage and molecular subtype, its direction remained consistent. CLU may therefore capture response-related biological information that partially overlaps with established clinicopathological factors, although its independent contribution requires confirmation in larger cohorts. For this reason and given the relatively limited number of patients with complete RCB data, these results should be interpreted as preliminary evidence requiring external validation. Nevertheless, the observed effect size, together with the excellent reproducibility of CLU immunohistochemical assessment, supports further investigation of CLU as part of multimarker predictive models integrating established clinicopathological and molecular variables. Rather than replacing current predictors, CLU may contribute complementary biological information regarding tumor sensitivity to systemic treatment. This concept is consistent with recent investigations indicating that future prediction of neoadjuvant chemotherapy response will likely rely on integrated clinicopathological and molecular biomarker models rather than individual biomarkers alone [5,7]. If validated, CLU assessment at diagnosis could contribute to identifying patients less likely to achieve a substantial pathological response and complement established clinicopathological factors in future response-stratification models.

In contrast, CLU expression at diagnosis was not associated with disease-free survival, distant recurrence-free survival or overall survival, suggesting that CLU primarily reflects initial chemosensitivity rather than long-term prognosis. These findings are also consistent with our previous observations in a large breast carcinoma series, in which CLU expression, despite increasing along tumor progression, was not an independent prognostic indicator [16]. Such a dissociation is biologically plausible, because survival after neoadjuvant chemotherapy is shaped by many factors beyond the initial pathological response, including subsequent systemic therapies, tumor evolution and acquired resistance; biomarkers associated with treatment response are therefore not necessarily expected to retain independent prognostic value over long-term follow-up [4]. Tumor aggressiveness and chemoresistance are closely intertwined but do not necessarily evolve in parallel. Highly proliferative tumors may be particularly sensitive to cytotoxic therapy, while genomic instability, cellular plasticity and stress adaptation can promote the emergence of resistant subclones [20]. This paradox is well illustrated by triple-negative breast cancer, which combines high rates of pathological complete response with poor outcomes among patients with residual disease. In this context, the behavior of CLU is particularly informative: higher CLU expression was inversely associated with histological grade yet associated with greater residual disease burden, while carrying no prognostic information. Consistent with its cytoprotective and stress-adaptive functions, CLU may therefore reflect the capacity to survive treatment rather than baseline proliferative aggressiveness.

This study has several strengths. To our knowledge, this is one of the first multicenter studies evaluating CLU protein expression in routine pretreatment diagnostic biopsies using RCB as the primary pathological response endpoint. It was conducted in a multicenter cohort with long-term clinical follow-up, and CLU expression was assessed centrally using a standardized immunohistochemical protocol with excellent interobserver reproducibility, supporting the feasibility of its assessment in routine diagnostic pathology. In addition, all analyses were predefined according to the available pathological response systems, avoiding unnecessary harmonization of biologically distinct response classifications.

Several limitations should be acknowledged. This retrospective study evaluated CLU as a single biomarker, and quantitative RCB was available only in a subset of patients due to its progressive implementation across centers and over time. This reduced statistical power and, given differences in subtype, center and diagnosis year between included and non-included patients, potential selection bias cannot be excluded. A small proportion of patients (2–5%) lacked ascertainable event dates, and the sample size limited subgroup and interaction analyses. CLU scoring combined cytoplasmic, membranous and stromal staining, precluding a dedicated epithelial-versus-stromal analysis, although no case showed isolated stromal positivity. Treatment heterogeneity over the long recruitment period, including evolving HER2-directed therapy, could not be accounted for. External validation in larger, contemporary cohorts, ideally incorporating CLU into integrated predictive models, is therefore warranted.

In conclusion, higher CLU expression in pretreatment diagnostic breast biopsies was associated with greater residual burden following neoadjuvant chemotherapy. Although its independent or incremental predictive value was not demonstrated in this cohort, the reproducibility of CLU immunohistochemical assessment and its association with RCB support further evaluation of CLU as a candidate response-associated marker that would require validation before it could inform treatment decisions.

## Figures and Tables

**Table 1:** Baseline clinicopathological and molecular characteristics of the study cohort.

Characteristic	*n* (%)
Patients	152 (100)
Age at diagnosis, years, mean ± SD	50.21 ± 11.08
Family history	
- Absence	107 (70.4)
- Presence	45 (29.6)
Hormonal status	
- Pre/perimenopausal	84 (55.3)
- Menopausal	68 (44.7)
Diagnostic context	
- Radiological	24 (15.8)
- Clinical	128 (84.2)
Histological type	
- Ductal	138 (90.8)
- Lobular	13 (8.6)
- Tubular	1 (0.7)
Histological grade	
- Grade I	8 (6.8)
- Grade II	49 (41.5)
- Grade III	61 (51.7)
Missing/not determined	34
Clinical stage	
- Stage I	11 (8.2)
- Stage IIA	24 (17.9)
- Stage IIB	42 (31.3)
- Stage IIIA	31 (23.1)
- Stage IIIB	18 (13.4)
- Stage IV	8 (6.0)
Missing/not determined	18
Immunophenotype	
- Luminal A	14 (9.2)
- Luminal B	70 (46.1)
- Luminal A or Luminal B	7 (4.6)
- Luminal B HER2	18 (11.8)
- HER2-enriched	9 (5.9)
- Basal-like	34 (22.4)
Estrogen receptor	
- Positive	102 (67.1)
- Negative	50 (32.9)
Progesterone receptor	
- Positive	99 (65.1)
- Negative	53 (34.9)
HER2	
- Positive	37 (26.1)
- Negative	105 (73.9)
Missing/not determined	10
Ki67	
- <20%	32 (21.3)
- ≥20%	118 (78.7)
Missing	2
TILs	
- Low (<10%)	70 (54.7)
- Intermediate (10–40%)	42 (32.8)
- High (>40%)	16 (12.5)
Missing	24
CLU expression	
- Negative	31 (23.7)
- 10–50%	35 (26.7)
- 50–90%	26 (19.8)
- >90%	39 (29.8)
Missing/not evaluable	21

Data are presented as *n* (%) unless otherwise indicated. Percentages were calculated using the number of evaluable cases for each variable as the denominator; missing or not determined values were excluded from percentage calculations and are reported separately. Age is presented as mean ± standard deviation (SD). Abbreviations: CLU, clusterin; HER2, human epidermal growth factor receptor 2; SD, standard deviation; TILs, tumor-infiltrating lymphocytes.

**Table 2:** Association between CLU expression and pathological response to neoadjuvant chemotherapy. (**A**) Association between CLU expression and residual cancer burden (full cohort assessable for RCB, *n* = 73). (**B**) Pathological complete response according to CLU expression (full cohort assessable for pCR, *n* = 105). (**C**) Univariable analyses of the association between CLU expression and pathological response. (**D**) Multivariable ordinal logistic regression for RCB ^1^.

**(A)**						
	**RCB According to Four CLU Expression Categories**				**RCB According to Low Versus High CLU Expression**	
RCB	Negative *n* = 10	10–50% *n* = 18	50–90% *n* = 21	>90% *n* = 24	<50% *n* = 28	≥50% *n* = 45
RCB 0	4 (40.0)	6 (33.3)	2 (9.5)	2 (8.3)	10 (35.7)	4 (8.9)
RCB I	2 (20.0)	0 (0.0)	4 (19.0)	5 (20.8)	2 (7.1)	9 (20.0)
RCB II	2 (20.0)	8 (44.4)	8 (38.1)	8 (33.3)	10 (35.7)	16 (35.6)
RCB III	2 (20.0)	4 (22.2)	7 (33.3)	9 (37.5)	6 (21.4)	16 (35.6)
**(B)**						
**CLU Expression**			***n***		**pCR, *n* (%)**	
Negative			22		6 (27.3)	
10–50%			27		8 (29.6)	
50–90%			24		3 (12.5)	
>90%			32		4 (12.5)	
CLU < 50%			49		14 (28.6)	
CLU ≥ 50%			56		7 (12.5)	
**(C)**						
**Analysis**					**OR (95% CI)**	***p***
Four-level ordinal CLU model ^2^					1.53 (1.01–2.31)	0.044
RCB distribution, CLU <50% vs. ≥50% ^3^					—	0.023
RCB 0, CLU <50% vs. ≥50%, Firth model ^4^					5.23 (1.53–17.96)	0.008
pCR trend across four CLU categories (Cochran-Armitage) ^5^						0.075
pCR, CLU <50% vs. ≥50% ^6^					2.80 (1.02–7.65)	0.051
**(D)**						
**Variable**			**Adjusted OR (95% CI)**			***p*** **-Value**
CLU, per category increase			1.34 (0.86–2.07)			0.194
Age, per year			1.00 (0.95–1.04)			0.845
Stage III–IV vs. I–II			4.10 (1.50–11.23)			0.006
HR+/HER2− vs. HER2+			10.42 (3.22–33.79)			<0.001
TNBC vs. HER2+			1.72 (0.42–7.04)			0.448

Data are presented as *n* (%) unless otherwise indicated. RCB analyses were based on 73 evaluable patients, pCR analyses on 105 evaluable patients, and the multivariable RCB model on 68 patients with complete covariate data. Low CLU expression was defined as <50% and high CLU expression as ≥50%. ^1^ Multivariable ordinal logistic regression evaluating RCB category (0–III) among patients with complete covariate data (*n* = 68). The model included CLU expression as an ordinal variable, age as a continuous variable, clinical stage dichotomized as I–II versus III–IV, and breast cancer subtype categorized as HR+/HER2−, HER2-positive, or triple-negative, with HER2-positive disease as the reference category. ^2^ Ordinal logistic regression evaluating the ordered association between the four CLU expression categories and RCB 0–III. The proportional-odds assumption showed no evidence of violation (*p* = 0.265). ^3^ Overall comparison of RCB distribution between low and high CLU expression groups using the χ^2^ test. ^4^ Penalized Firth logistic regression comparing the odds of achieving RCB 0 in tumors with CLU <50% versus CLU ≥50%. ^5^ Cochran–Armitage test for linear trend in pCR across the four CLU expression categories. ^6^ pCR frequencies compared between low (<50%) and high (≥50%) CLU tumors; OR and 95% CI estimated by unconditional logistic regression (Wald-based interval); *p* value from Fisher’s exact test. The Wald interval and the exact test rely on different approximations and may diverge near the 0.05 threshold in small, sparse tables. Abbreviations: CI, confidence interval; CLU, clusterin; OR, odds ratio; RCB, residual cancer burden. —, not applicable; this analysis compares the overall RCB distribution using the chi-square test and does not provide an odds ratio.

**Table 3:** Association between CLU expression and long-term oncological outcomes. Time-to-event analyses.

Outcome	*n*	Events	HR per Increase in CLU Category (95% CI)	*p*-Value
DFS	128	47	0.95 (0.73–1.23)	0.702
DRFS	129	44	0.88 (0.67–1.16)	0.359
OS	124	37	1.04 (0.76–1.42)	0.794

Cox proportional-hazards models for time-to-event outcomes. Hazard ratios represent the change in hazard for each increase in CLU expression category (negative, 10–50%, 50–90%, and >90%). Abbreviations: CI, confidence interval; CLU, clusterin; DFS, disease-free survival; DRFS, distant recurrence-free survival; HR, hazard ratio; OS, overall survival.

## Data Availability

The data that support the findings of this study are available from the corresponding author upon reasonable request.

## Supplementary Materials

The following supporting information can be downloaded at: https://www.mdpi.com/article/10.3390/jcm15197557/s1, Table S1. Baseline clinicopathological characteristics of patients included and not included in the CLU–RCB analysis.

## Abbreviations

The following abbreviations are used in this manuscript:

CLU	Clusterin, APO-J
CI	Confidence Interval
DFS	Disease-Free Survival
DRFS	Distant Recurrence-Free Survival
ER	Estrogen Receptor
FFPE	Formalin-Fixed Paraffin-Embedded
HER2	Human Epidermal Growth Factor Receptor 2
HR	Hormone Receptor
IHC	Immunohistochemistry
OR	Odds Ratio
OS	Overall Survival
pCR	Pathological Complete Response
sTILs	Stromal Tumor Infiltrating Lymphocytes
TNBC	Triple Negative Breast Cancer
