# Supplementary material for: Tumor Clusterin Expression at Diagnosis Is Associated with Residual Disease Burden After Neoadjuvant Chemotherapy in Breast Cancer
Source: J Clin Med. 2026 Sep 29;15(19):7557. doi: 10.3390/jcm15197557 (PMC13665789; doi:10.3390/jcm15197557)
Supplement: Supplementary file 1 [file jcm-15-07557-s001.zip › jcm-4580126-supplementary.pdf]

Supplementary material

Supplementary Table S1. Baseline clinicopathological characteristics of patients included and not included in the CLU–RCB analysis.

**Characteristic**	**Included in CLU–RCB analysis (n=73)**	**Not included (n=79)**	***p*-value**
Age at diagnosis, median (years)	51	47	0.053
Clinical stage			1.000
I–II	39/68 (57.4%)	38/66 (57.6%)	
III–IV	29/68 (42.6%)	28/66 (42.4%)	
Histological grade			0.360
Grade 1	4/62 (6.5%)	4/56 (7.1%)	
Grade 2	29/62 (46.8%)	19/56 (33.9%)	
Grade 3	29/62 (46.8%)	33/56 (58.9%)	
ER-positive	50/73 (68.5%)	52/79 (65.8%)	0.734
PR-positive	46/73 (63.0%)	52/79 (65.8%)	0.737
Ki-67 ≥20%	59/73 (80.8%)	63/77 (81.8%)	1.000
Breast cancer subtype			**<0.001**
HR+/HER2−	40/73 (54.8%)	50/79 (63.3%)	
HER2+	22/73 (30.1%)	5/79 (6.3%)	
TNBC	11/73 (15.1%)	24/79 (30.4%)	
Participating center			**<0.001**
HCS	43/73 (58.9%)	19/79 (24.1%)	
HUVR	29/73 (39.7%)	25/79 (31.6%)	
HUVM	1/73 (1.4%)	28/79 (35.4%)	
HUSC	0/73 (0%)	7/79 (8.9%)	
Year of diagnosis, median	2016	2014	**<0.001**

Data are presented as n/N (%) unless otherwise indicated. Percentages were calculated using available data for each variable. Missing or undetermined values were excluded from the corresponding comparison. Categorical variables were compared using χ^2 or Fisher's exact test, as appropriate; age and year of diagnosis were compared using non-parametric tests.
